# Preventive Treatment of Yellow Fever in Havana

**Published:** 1878-09

**Authors:** 


Preventive Treatment of Yellow Fever in Havana. Leaving for the moment the great questions of quarantine and disinfection, let us speak of several indications or advices for people, who come from countries, where black vomit or yellow fever does not exist epidemically. We say so, because we have often heard people from New Orleans saying: "We are not afraid of yellow fever in Havana, it exists sometimes in our country, in consequence we are not liable to have it." This is a fatal mistake that has cost many lives. There is no doubt that the inhabitants of those places, or others similar are less subject to yellow fever, but between this and a total immunity there is a great distance. We will say that every person of any class, age, or profession who comes to Havana is liable to contract yellow fever, and with the less security, as their nationalties are more distant from the regions limited by the Mexican Gulf. We want to destroy a fatal prejudice, that has been the cause of many victims, on account of the neglect, with which several fevers in women and children are looked upon, believing that they cannot take the black vomit.

The first thing that a foreigner has to do, immediately after landing, is to locate himself in one of the healthiest places of the city; to dress, and modify his ordinary habits of eating and drinking, according to the usages of the country: to stay in the house during the hottest hours of the day, that is between 11 A. M. and 3 P. M.; to be moderate in both physical and intellectual works; to take every once in a while saline purgatives, and luke warm baths; not to sleep with windows open during the night; to avoid any kind of excess, as nervous excitement is very prejudicial, and favorable to the development of the disease. Indigestions are particularly terrible.

They can drink lemonades and orangeades without the abuse of ice, which is highly irritant when used in excess. They must also be careful about taking cool refrigerating drinks before the digestion be effected.

Good and well ripened fruits are not dangerous, no matter what the vulgus says against them.

The use of cotton undershirts is very healthy, by keeping away that disagreeable chilly sensation caused by the contact of linen shirts damped by excessive perspiration. If in spite of these precautionary measures, he feels some symptoms of disease, he must not lose time, but send immediately after a physician, because yellow fever is one of the diseases, that requires the greatest activity in its treatment. Six or seven hours lost are sufficient to compromise the cure, or at least to bring accidents that could have been avoided. It is a disease that we can cut off in the commencement. Let people take note of this.

The physician, who is called thirty or forty hours after the invasion of the fever is perplexed, as congestive disorders already exist, and albumen is abundant in the urine, and he is no longer able to avoid stomachal and intestinal hæmorrhages, which a proper treatment commenced several hours before could have prevented.

At the hour we are writing this paper, a child five years and a half old is dying, who fell sick on the 21st. Its parents, convinced that children are exempt from yellow fever, waited to call us until the 23d, forty-eight hours afterwards. At our fourth visit, intestinal hæmorrhages made their appearance, and the child died on the eighth day of the disease with tetanic symptoms. Seeing such a vital resistance in this patient, it is more than probable, that he could have recovered, had the medical treatment been initiated in the first hour of the disease.

The indications, that we have just suggested, are only hygienical; we believe, that the public has the right to claim from us something more. We have thought of it, and we are going to propose a preparation composed of quinine and extract of nux vomica.

We believe, that in times of epidemic, it would be good to take every morning according to age, or sex, from ten to twenty-five centigrammes of either sulphate or bromhydrate of quinine with two or three centigrammes of nux vomica, either in pills, or dissolved in a little water or coffee. We cannot assure that this prescription really prevents yellow fever, but we would not be surprised, that if for some people it might be a preventative ; for others who fall sick in spite of its use, might be a means of moderating the violence of the symptoms, and converting into a fever of acclimation, cases that otherwise would have been genuine yellow fever. We are often consulted by the new immigrants to this Island whether blood letting from the arm be a good preventive against the fever. We answer that in plethoric persons we think it useful, and in proof of it we will relate the following fact :

Thirty years ago there was in Santiago de Cuba, a physician who had charge during many years of the barrack troops. He used to send acclimated soldiers to a very fresh and elevated place, called El Morro, situated at the entrance of the harbor. He bled them from the arm, once or twice ; he administered a couple of saline purgatives to each soldier, and sent them back to the city fifteen or twenty days afterwards. An old friend and confrere of ours told us that black vomit was in those times almost unknown among the troops.

Here ends our work. It is an exact epilogue of what we have seen, exempt from all influence of school, and without any preconceived idea. By giving this preventive treatment based upon remarkable success, we have been guided by good faith, and by the desire of being useful ; we are always ready to acknowledge our errors, and to accept anything better, that the field of scientific truth may afford us.

Before leaving our readers, and colleagues, we want to call the special attention of the profession to the following points :

1st. To determinate the precise moment, when albumen appears in the urine. 2d. To try the influence of oxygen mixed with the air, that the patient breathes. 4th. To examine the condition of the blood by means of polarization, according to the late magnificent works of Fumouge. 5th. To investigate with great care the internal lesions through pathological anatomy: it is a void in the history of yellow fever, that could well be filled by the intelligent professors, that are at the head of the civil and military hospitals of this city.—*New Orleans Medical and Surgical Journal.*

